# Translating the promise of 5HT_4_ receptor agonists for the treatment of depression

**DOI:** 10.1017/S0033291720000604

**Published:** 2021-05

**Authors:** Susannah E Murphy, Angharad N de Cates, Amy L Gillespie, Beata R Godlewska, Jessica C Scaife, Lucy C Wright, Philip J Cowen, Catherine J Harmer

**Affiliations:** 1 University Department of Psychiatry, Warneford Hospital, University of Oxford, OX3 7JX, UK; 2 Oxford Health NHS Foundation Trust, Warneford Hospital, Oxford, UK

**Keywords:** Antidepressants, cognition, emotion, serotonin

## Abstract

Animal experimental studies suggest that 5-HT_4_ receptor activation holds promise as a novel target for the treatment of depression and cognitive impairment. 5-HT_4_ receptors are post-synaptic receptors that are located in striatal and limbic areas known to be involved in cognition and mood. Consistent with this, 5-HT_4_ receptor agonists produce rapid antidepressant effects in a number of animal models of depression, and pro-cognitive effects in tasks of learning and memory. These effects are accompanied by molecular changes, such as the increased expression of neuroplasticity-related proteins that are typical of clinically useful antidepressant drugs. Intriguingly, these antidepressant-like effects have a fast onset of their action, raising the possibility that 5-HT_4_ receptor agonists may be a particularly useful augmentation strategy in the early stages of SSRI treatment. Until recently, the translation of these effects to humans has been challenging. Here, we review the evidence from animal studies that the 5-HT_4_ receptor is a promising target for the treatment of depression and cognitive disorders, and outline a potential pathway for the efficient and cost-effective translation of these effects into humans and, ultimately, to the clinic.

## Introduction

The potentiation of monoamine activity, and in particular serotonergic neurotransmission, has been the dominant target of antidepressant treatments since the serendipitous discovery of the antidepressant effects of monoamine oxidase inhibitor and tricyclics in the 1950s. Whilst drugs such as selective serotonin reuptake inhibitors (SSRIs) are more effective than placebo (Cipriani et al., 2018), there are significant limitations to current treatment approaches. Only about one third of patients achieve remission after 14 weeks of SSRI treatment and around 30% of patients remain ‘treatment resistant’, even after trying multiple treatment approaches (Rush et al., 2009; Trivedi et al., 2006). When SSRIs are effective, they require chronic administration to achieve therapeutic efficacy and it can take several weeks for depressive symptoms to fully resolve (Frazer & Benmansour, 2002; Mitchell, 2006). SSRI treatment is also associated with adverse effects which can compromise treatment adherence, including gastrointestinal symptoms, drowsiness, weight gain, sexual dysfunction and emotional blunting (Goethe, Woolley, Cardoni, Woznicki, & Piez, 2007; Goodwin, Price, De Bodinat, & Laredo, 2017). Given that depression is now ranked as the single largest contributor to global disability (World Health Organisation, 2017), there is a pressing need for novel treatment approaches that are more effective, better tolerated and faster acting.

SSRIs selectively block serotonin (5-hydroxytryptamine, 5-HT) transporters, which results in an increase in 5-HT levels at all post-synaptic serotonin receptor subtypes. This indirect activation of a broad range of receptors is likely to contribute not only to the therapeutic action, but also to the unwanted aspects of SSRIs. In particular, early activation of 5-HT_1A_ autoreceptors by SSRIs results in a paradoxical decrease in 5-HT cell firing, and is thought to contribute to the slow onset of the clinical action of SSRIs (Artigas, 2013). Equally, activation of post-synaptic 5-HT_2C_ receptors has been associated with the increase in anxiety that can characterise initiation of SSRI treatment (Burghardt, Bush, McEwen, & LeDoux, 2007).

More selective activation of specific 5-HT receptors has the potential to produce better tolerated, faster acting treatments. Preclinical studies suggest that the 5-HT_4_ receptor is a particularly promising therapeutic target for this approach. In animal models, 5-HT_4_ receptor activation has antidepressant effects across a range of behavioural paradigms. Intriguingly, the onset of antidepressant effects following 5-HT_4_ receptor agonism is more rapid than that seen with SSRIs, and is accompanied by early molecular and cellular changes that mirror those seen following chronic administration of conventional antidepressants. This profile of effects suggests that 5-HT_4_ receptor agonists may hold potential as novel antidepressants, and may be useful adjuncts to SSRI treatment to increase the speed of onset of therapeutic effects. In addition, 5-HT_4_ receptor agonists produce pro-cognitive effects in tasks of learning and memory in rodents, highlighting a potential role in targeting cognitive deficits associated with depression, which are not well addressed by current antidepressant medications (Shilyansky et al., 2016).

To date, the evidence that the 5-HT_4_ receptor might be a useful treatment target mainly comes from preclinical studies, and it is important that these findings are translated into human studies. Human PET imaging studies using the [^11^C]-SB 207145 ligand have provided some initial support for a role of the 5-HT_4_ receptor in the pathophysiology of depression (Madsen et al., 2014). However, it remains to be established whether activation of 5-HT_4_ receptors in humans has effects that mirror the antidepressant and pro-cognitive effects seen in animals, and therefore whether this is a viable antidepressant target. Until recently, such experimental studies in humans have not possible due to a lack of suitable compounds. With the licensing of the 5-HT_4_ partial receptor agonist, prucalopride (Resolor), for the treatment of constipation, there is a timely opportunity to establish the effects of 5-HT_4_ activation on mood and cognition in humans, using an experimental medicine framework. Here, we review the evidence from animal studies that the 5-HT_4_ receptor is a promising target for the treatment of depression and cognitive disorders, and outline a potential pathway for the efficient translation of these effects into humans and, ultimately, to the clinic.

## 5-HT_4_ receptors are expressed in the basal ganglia and limbic system

Since its first description 30 years ago (Dumuis, Bouhelal, Sebben, Cory, & Bockaert, 1988), the 5-HT_4_ receptor has been discovered both in the brain and in various sites in the periphery. Peripherally, 5-HT_4_ receptors are found in the heart (Kaumann, 1990), the intestine (Craig & Clarke, 1990), the adrenal glands (Idres, Delarue, Lefebvre, & Vaudry, 1991) and the bladder (Tonini & Candura, 1996). In the brain, 5-HT_4_ receptors are located post-synaptically and are expressed in the hypothalamus, hippocampus, basal ganglia, amygdala, olfactory bulb, septal area and neocortex (Cai, Flores-Hernandez, Feng, & Yan, 2002; Compan et al., 1996; Roychowdhury, Haas, & Anderson, 1994; Vilaró, Cortés, & Mengod, 2005; Waeber, Sebben, Nieoullon, Bockaert, & Dumuis, 1994). In rodents, 5-HT_4_ receptors are mainly situated on gamma-aminobutyric acid (GABA) neurones in the striatum and nucleus accumbens (Compan et al., 1996), glutamate pyramidal cells in the medial prefrontal cortex (PFC) and hippocampus (Cai et al., 2002; Vilaró et al., 2005) and hippocampal granule cells (Vilaró et al., 2005). 5-HT_4_ receptors have also been described in the rat dorsal raphe nucleus (DRN) in autoradiographic studies (Vilaró et al., 2005; Waeber et al., 1994), although 5-HT_4_ receptor mRNA has not been found suggesting that the 5-HT_4_ receptors are located on DRN afferent neurons rather than on DRN 5-HT cells (Vilaró et al., 2005).

Evidence from PET studies in humans confirms that 5-HT_4_ receptors have a high density in the caudate, putamen and nucleus accumbens, with lower expression across a broad range of cortical and limbic areas, including the hippocampus and amygdala (Beliveau et al., 2017). Similarly, the highest levels of human 5-HT_4_ receptor mRNA have been found in the caudate and putamen, with lower levels in the amygdala temporal cortex and hippocampus (Medhurst, Lezoualc'h, Fischmeister, Middlemiss, & Sanger, 2001). Post-mortem human brain studies have also confirmed a high density of the 5-HT_4_ receptor in the basal ganglia (caudate nucleus, putamen, nucleus accumbens, globus pallidus and substantia nigra), as well as the hippocampus (particularly in the CA1 region) and an intermediate density in the neocortex and amygdala (Bonaventure et al., 2000; Varnas, Halldin, Pike, & Hall, 2003). In addition, low levels of 5-HT_4_ binding sites have been reported in the human thalamus and raphe nuclei (Beliveau et al., 2017; Varnas et al., 2003).

## The neurochemical and molecular effects of 5-HT_4_ receptor activation

5HT_4_ receptors are transmembrane-spanning G protein-coupled receptors, and their activation results in increased neuronal excitability that is mediated through the stimulation of adenylyl cyclase and an increase in cyclic adenosine monophosphate (Bockaert, Claeysen, Compan, & Dumuis, 2008; Dumuis et al., 1988). In addition, 5-HT_4_ receptor activation initiates important G protein-independent pathways, including the activation of tyrosine kinase Src and, in turn, neuronal extracellular-signal-regulated kinase (ERK) (Barthet et al., 2007), which is known to play a critical role in hippocampal synaptic plasticity (Norman, Thiels, Barrionuevo, & Klann, 2000).

5-HT_4_ receptors also indirectly affect neuronal function through their modulation of neurotransmitter release, including GABA (Bijak & Misgeld, 1997), acetylcholine (Consolo, Arnaboldi, Giorgi, Russi, & Ladinsky, 1994; Johnson et al., 2012; Siniscalchi, Badini, Beani, & Bianchi, 1999), dopamine (Bockaert, Claeysen, Compan, & Dumuis, 2004; Bockaert et al., 2008; Bonhomme, De Deurwaerdere, Le Moal, & Spampinato, 1995), histamine (Johnson et al., 2012) and serotonin (Ge & Barnes, 1996; Licht et al., 2010). Of particular interest is the enhanced release of acetylcholine in the frontal cortex and hippocampus following 5-HT_4_ receptor activation, which has been linked to its facilitatory role in memory and cognition (Hagena & Manahan-Vaughan, 2017; King, Marsden, & Fone, 2008). In support of this 5-HT_4_ receptor agonists have been shown to increase electrically-evoked [3H]choline efflux in guinea pig brain slices in the cerebral cortex, hippocampus and nucleus basalis magnocellularis (NbM) (Siniscalchi et al., 1999), and facilitate the release of ACh selectively in the frontal cortex (Consolo et al., 1994). Importantly, both of these effects are blocked by 5-HT_4_ receptor antagonists, which further supports a specific role for the 5-HT_4_ receptor in cholinergic neurotransmission. Given that 5-HT_4_ receptors are not present on cholinergic basal forebrain neurons, it is thought that the enhancement of ACh release induced by 5-HT_4_ receptor agonists is likely to be mediated by GABAergic and/or glutamatergic cell populations (Penas-Cazorla & Vilaro, 2015).

Importantly, 5-HT_4_ receptors in the PFC control the firing rate of midbrain serotonergic cells. Consistent with this, the basal firing of 5-HT cells in the DRN is reduced by over 50% in 5-HT_4_ receptor knockout mice compared with wildtypes (Conductier et al., 2006). The facilitatory control exerted by 5-HT_4_ receptors on serotonergic firing is thought to operate via a positive PFC–DRN feedback loop in which the activation of postsynaptic 5-HT_4_ receptors located on medial PFC pyramidal cells has an excitatory effect on serotonin neurons in the DRN dorsal raphe, resulting in an increase in the release of serotonin in target structures such as the PFC and hippocampus. Consistent with this model, overexpression of 5-HT_4_ receptors in the mPFC, but not in the striatum or hippocampus, increases DRN 5-HT neuronal activity (Lucas et al., 2005). In addition, pharmacological studies have demonstrated that administration of the selective 5-HT_4_ receptor agonists, RS67333 and prucalopride, increases DRN 5-HT cell firing (Faye et al., 2019; Lucas et al., 2005; Lucas & Debonnel, 2002; Moha ou Maati et al., 2016). Intriguingly, this increase in firing can be seen within 30 min of drug administration, suggesting that 5-HT_4_ receptor agonists hold potential as rapid onset antidepressant agents (Lucas et al., 2005; Lucas & Debonnel, 2002). This is in stark contrast to the decrease in raphe cell firing that is characteristic of SSRIs, and which has been implicated in their delayed onset of action (Blier, Piñeyro, el Mansari, Bergeron, & de Montigny, 1998). Importantly, the excitatory effect of 5-HT_4_ receptor agonists on the firing of midbrain 5-HT cells is abolished by bilateral electrolytic lesions of the mPFC, demonstrating a causal role of the mPFC in the facilitation of 5-HT cell firing by 5-HT_4_ receptor agonists (Moha ou Maati et al., 2016).

Short-term 5-HT_4_ receptor activation has also been shown to increase hippocampal cell proliferation and the expression of neuroplasticity-related proteins such as CREB and BDNF (Ishizuka, Goshima, Ozawa, & Watanabe, 2014; Lucas et al., 2007; Pascual-Brazo et al., 2012). These mirror those seen with chronic antidepressant administration (Malberg, Eisch, Nestler, & Duman, 2000; Warner-Schmidt & Duman, 2006), but occur more rapidly. For example, 3 days administration of the 5-HT_4_ receptor agonist, RS67333, increases hippocampal cell proliferation in the dentate gyrus and the expression of neuroplasticity-related proteins, such as BDNF and CREB (Lucas et al., 2007; Pascual-Brazo et al., 2012). After 7 days of administration the level of these neuroplastic changes are comparable with those seen after 2–3 weeks of SSRI administration (Malberg et al., 2000; Pascual-Brazo et al., 2012; Samuels et al., 2016). Further, long-term 5-HT_4_ receptor activation (4 weeks) facilitates the maturation of newborn neurons in the adult rat hippocampus (Mendez-David et al., 2014).

Furthermore, 5-HT_4_ receptors are involved in fine-tuning of the key cellular processes underlying synaptic plasticity, long-term potentiation (LTP) and long-term depression (LTD), which is likely to play a key role in their effects on long term hippocampal-dependent memory. Activation of the 5-HT_4_ receptor has been shown to prevent LTD in all the main hippocampal subfields (Kemp & Manahan-Vaughan, 2005; Twarkowski, Hagena, & Manahan-Vaughan, 2016). The effect of 5-HT_4_ receptor activation on LTP is complex and specific to individual hippocampal subfields, which may relate to differences in the expression of different receptor isoforms (Hagena & Manahan-Vaughan, 2017). A number of studies have demonstrated that stimulation of 5-HT_4_ receptors in freely behaving rats promotes synaptic information-encoding through LTP at both the CA1 region (Kemp & Manahan-Vaughan, 2005) and dentate gyrus (Kulla & Manahan-Vaughan, 2002; Twarkowski et al., 2016). Conversely, LTP at mossy fibre synapses of the CA3 region is suppressed by 5-HT_4_ receptor activation (Twarkowski et al., 2016). The effect of 5-HT_4_ receptor activation on LTP has also been shown to be time-dependent; for example, the long lasting sustainment of LTP (i.e. after 48 h) in the dentate gyrus can be impaired by 5-HT_4_ receptor stimulation (Twarkowski et al., 2016). Taken together, this pattern suggests that 5-HT_4_ receptor activation acts to promote information encoding through LTP in CA1 and dentate gyrus at the expense of information coding through LTD, which is likely to impact upon the content of information encoding (Hagena & Manahan-Vaughan, 2017; Twarkowski et al., 2016). The effect of 5-HT_4_ receptor activation on dentate gyrus plasticity is particularly interesting in light of recent evidence that LTP induction in the dentate gyrus is sufficient to induce antidepressant-like effects in rat models within a rapid timeframe (Kanzari et al., 2018), suggesting that the facilitatory effect of 5-HT_4_ receptor agonists on DG plasticity may, at least in part, account for their fast-acting antidepressant-like properties.

## Preclinical studies support a role for the 5-HT_4_ receptor in the aetiology of depression and anxiety

Preclinical studies provide support for the involvement of the 5-HT_4_ receptor in the aetiology of depression. Changes in 5-HT_4_ receptor expression have been reported in a number of rodent models of depression, although the direction of effect varies by model. In the olfactory bulbectomy (OBX) and glucocorticoid receptor heterozygous [GR(±)] models, an increase in 5-HT_4_ receptor binding has been reported in the ventral hippocampus (OBX) and striatum [GR(±)] (Licht et al., 2010). In contrast, in the Flinders-sensitive line rat model of depression, a decrease in hippocampal 5-HT_4_ receptor binding was reported (Licht et al., 2009). 5-HT_4_ receptor mRNA and protein has also been shown to be down-regulated in rats subjected to maternal deprivation (but not chronic unpredictable stress), with a strong correlation between levels of hippocampal 5-HT_4_ receptor mRNA and anhedonic-like behaviour in these animals (Bai et al., 2014).

5-HT_4_ receptor knock out mice do not show a clear depressed-like phenotype, with no differences in behaviour on the forced swim test, which may be due to the considerable adaptations in the serotoninergic system that occur across development in these animals (Amigo et al., 2016; Conductier et al., 2006). However, the knock out animals do show some evidence of anhedonic- and apathetic-like behaviour, with a reduction in sucrose consumption and nesting behaviour, and decreased central time in the open field (OF), which is suggestive of increased anxiety (Amigo et al., 2016). Interestingly, a recent study suggests that focal overexpression of the 5-HT_4_ receptor in the medial PFC produces an ‘anti-depressed’ and anxiolytic behavioural phenotype, which is consistent with the central role ascribed to descending inputs from the mPFC to the DRN in mediating increased raphe firing (Castello et al., 2018).

## 5-HT_4_ receptors are involved in the mechanism of action of SSRIs

There is also convincing evidence that 5-HT_4_ receptors play a role in the action of SSRIs in animal models. Chronic administration of monoaminergic antidepressants, such as fluoxetine, paroxetine and venlafaxine (but not reboxetine), decreases 5-HT_4_ receptor density in the basal ganglia and hippocampus in the rat brain (Licht et al., 2009; Vidal, Valdizan, Mostany, Pazos, & Castro, 2009; Vidal, Valdizan, Vilaró, Pazos, & Castro, 2010). In contrast, expression of the 5-HT_4_ receptor has been shown to be upregulated after chronic fluoxetine administration in corticostriatal pyramidal cells expressing the adaptor protein p11, located in layer 5 of the cerebral cortex (Schmidt et al., 2012).

More direct evidence for a role for the 5-HT_4_ receptor in the action of conventional antidepressants comes from studies in which 5-HT_4_ receptor ligands have been shown to modify the effects of monoaminergic antidepressants. For example, administration of the 5-HT_4_ receptor agonist RS67333 has been shown to augment the acute effect of paroxetine on extracellular serotonin levels in the rat ventral hippocampus (Licht et al., 2010). Perhaps the most convincing evidence comes from studies which demonstrate that disrupting 5-HT_4_ receptor function can abolish the behavioural effects of SSRIs. For example, the 5-HT_4_ receptor antagonist GR125487 blocks the effects of fluoxetine on a range of depression- and anxiety-related paradigms in a corticosterone model (CORT) of anxiety/depression (Mendez-David et al., 2014), suggesting that 5-HT_4_ receptor activation is necessary for the antidepressant and anxiolytic effects of SSRIs (although see Cryan and Lucki, 2000). A similar effect is found in 5-HT_4_ receptor knock out mice, where chronic fluoxetine administration has been shown to reverse the behavioural effects of OBX in wild type but not knock out animals (Amigo et al., 2016).

Interestingly, there is also evidence to suggest that the 5-HT_4_ receptor contributes to the neurotrophic effects of chronic SSRI treatment. For example, the usual increase in cell proliferation in the dentate gyrus following chronic administration of fluoxetine is not present in 5-HT_4_ receptor knock out mice (Imoto et al., 2015), and is partially blocked by 5-HT_4_ receptor antagonism (Mendez-David et al., 2014), suggesting that the neurogenic effects of SSRIs may be partly mediated by the 5-HT_4_ receptor. Further support for this comes from a study which demonstrates that the induction of immature like granule cells by chronic fluoxetine treatment (‘dematuration’) is blocked in 5-HT_4_ receptor knock-out mice (Kobayashi et al., 2010).

## Short-term administration of 5-HT_4_ receptor agonists has antidepressant and anxiolytic-like effects in animals

Studies in rodents have demonstrated that 5-HT_4_ receptor agonists exert antidepressant-like effects on behavioural paradigms that mirror those seen with conventional SSRIs, but with a more rapid onset of action (Lucas et al., 2007; Mendez-David et al., 2014; Pascual-Brazo et al., 2012). Using the classic forced swim paradigm, the 5-HT_4_ receptor partial agonists RS67333 and prucalopride have been shown to reduce immobility following acute (Lucas et al., 2007) and 3–5 day administration (Gomez-Lazaro et al., 2012; Pascual-Brazo et al., 2012). These effects parallel those seen with acute SSRI administration, which also induces a reduction in immobility on this task, although the effect of the 5-HT_4_ agonists was almost double that seen with acute citalopram (Lucas et al., 2007).

Short-term administration of 5-HT_4_ agonists also induces behavioural changes in paradigms that require chronic administration of SSRIs to shift. For example, 3 days administration of RS67333 was sufficient to increase sucrose consumption in the chronic mild stress model of depression (Lucas et al., 2007) and in CORT rats (Pascual-Brazo et al., 2012), and decrease hyperlocomotion in the OBX model (Lucas et al., 2007). Within 7 days of RS67333 a complete reversal of the effects of corticosterone on sucrose consumption was seen, paralleling the effect of 21-day treatment with fluoxetine (Pascual-Brazo et al., 2012), and suggesting a rapid reversal of anhedonic behaviour following 5-HT_4_ receptor agonism. These effects are consistent with the neurobiological adaptations that have been reported following 3 days of 5-HT_4_ receptor agonism, including desensitisation of 5-HT_1A_ autoreceptors, increased CREB phosphorylation and increased hippocampal cell proliferation (Lucas et al., 2007), and suggest that 5-HT_4_ receptor agonists have a unique, rapid-acting profile of effects.

Intriguingly, co-administration of 5-HT_4_ receptor agonists with SSRIs prevents the attenuation in raphe firing that is typically seen at the onset of SSRI administration (Lucas et al., 2010), suggesting that 5-HT_4_ receptor agonists may have promise as an augmentation strategy for SSRI treatment. In support of this proposal, co-administration of RS67333 and the SSRI citalopram led to a synergistic inhibition of hippocampal firing, while the same combination potentiated the ability of RS67333 to phosphorylate CREB in the hippocampus and reduce immobility in the forced swim test (Lucas et al., 2010).

5-HT_4_ receptor agonists have also been associated with rapid effects on anxiety. Seven days administration of RS67333 reduces latency to feed in the novelty suppressed feeding (NSF) paradigm, whereas 14 days administration of fluoxetine is required to see the same effect (Pascual-Brazo et al., 2012). A similar effect was seen in the mouse CORT model of anxiety/depression, where 7 day administration of RS67333, but not fluoxetine, reduced anxiety-related behaviour in the OF test and the elevated plus maze (EPM), although the effects of subchronic RS67333 on the NSF were not replicated in this model (Mendez-David et al., 2014). Interestingly, the effects of RS67333 on the OF and EPM tests were also evident in animals who had undergone hippocampal X-irradiation, suggesting that they are not dependent on hippocampal neurogenesis (Mendez-David et al., 2014).

Consistent with a fast onset of action, acute administration of RS67333 is sufficient to induce an anxiolytic-like effect on the elevated zero-maze (Bell, Duke, Gilmore, Page, & Begue, 2014), and reverse the anxiogenic effects of chronic cannabinoid exposure during adolescence (Abboussi et al., 2016). A recent report confirmed an anxiolytic-like effect of acute systemic infusion of RS67333 on the EPM, the OF test and the NSF test (Faye et al., 2019). Intriguingly, a similar profile was seen when RS67333 was directly infused into the mPFC, and the anxiolytic-like effects of RS67333 were attenuated by optogenetic inhibition of the mPFC terminals in the DRN, suggesting that direct activation of 5-HT_4_ receptors in the mPFC is responsible for the fast anxiolytic effects of 5-HT_4_ receptor agonists, via the mPFC-DRN circuit (Faye et al., 2019).

The effects of 5HT_4_ receptor antagonism on animal depression- or anxiety-like behaviour are less well established. Acute administration of the 5-HT_4_ receptor antagonists SDZ205-557, GR113808 and SB204070 does not influence anxiety-related behaviour in the light/dark choice test in mice (Costall & Naylor, 1997). Four weeks administration of the antagonist GR125487 has also been shown not to have an effect on behavioural models of anxiety in a corticosterone model of depression/anxiety, although the antagonist did block the effects of an SSRI in this model (Mendez-David et al., 2014). In contrast, two studies have reported an anxiolytic effect of acute administration of SB204070, GR113808 (Silvestre, Fernandez, & Palacios, 1996) and SB207266A (Kennett, Bright, Trail, Blackburn, & Sanger, 1997; Silvestre et al., 1996) on the EPM test. It is unclear what explains these contradictory findings, although there are differences in dosage, timing of antagonist administration and behavioural outcome measures used in the studies, which may be important.

## 5-HT_4_ Receptor activation has a facilitatory effect on rodent tests of learning and memory

A large body of evidence demonstrates a facilitatory effect of 5-HT_4_ receptor activation on rodent and primate behavioural tests of cognition, and in particular learning and memory (Hagena & Manahan-Vaughan, 2017; King et al., 2008). Early studies using relatively non-selective compounds, such as the mixed 5-HT_4_ receptor agonist/5-HT_3_ receptor antagonist BIMU1 demonstrated a facilitation of autoshaping (Meneses & Hong, 1997) and associative memory (Marchetti-Gauthier, Roman, Dumuis, Bockaert, & Soumireu-Mourat, 1997), which was attributed to 5-HT_4_ receptor activation since both effects were blocked by co-administration of a 5-HT_4_ receptor antagonist. Subsequent studies using more selective agonists confirmed that acute 5-HT_4_ receptor activation improves a wide of range of behavioural tests of learning and memory, including place recognition (Lamirault & Simon, 2001), object recognition (Lamirault & Simon, 2001; Levallet, Hotte, Boulouard, & Dauphin, 2009; Moser et al., 2002), delayed matching-to-sample (Terry et al., 1998), Morris water maze (Lelong, Dauphin, & Boulouard, 2001), delayed spontaneous alternation (Mohler et al., 2007) and olfactory association learning (Marchetti et al., 2004; Marchetti et al., 2011) tasks. The positive effect of 5-HT_4_ receptor agonism on object recognition is also seen after chronic (14 day) activation of the receptor (Quiedeville et al., 2015). Critically, 5-HT_4_ receptor antagonism has consistently been shown to block these agonist-induced pro-cognitive effects, confirming a specific role for the 5-HT_4_ receptor in learning and memory (Fontana, Daniels, Wong, Clark, & Eglen, 1997; Lamirault & Simon, 2001; Mohler et al., 2007; Moser et al., 2002). Whilst 5-HT_4_ receptor activation has particularly strong effects on hippocampal-dependent learning and memory, it also facilitates other forms of cognition, including attention (as measured by the five choice serial reaction time task, Hille, Bate, Davis, and Gonzalez, 2008) and social memory (Letty et al., 1997).

The timing of 5-HT_4_ receptor activation seems to be critical to the profile of effects on memory processes, with a particularly clear role for the receptor in learning and memory acquisition (Hagena & Manahan-Vaughan, 2017). Consistent with this, studies in which an agonist is administered prior to training have typically shown a facilitatory effect of 5-HT_4_ receptor activation (Lamirault & Simon, 2001; Meneses & Hong, 1997; Orsetti, Dellarole, Ferri, & Ghi, 2003), whereas the effect of post-training agonist administration is less clear, with reports of both enhanced (Lamirault & Simon, 2001; Orsetti et al., 2003) and impaired (Meneses & Hong, 1997; Nasehi, Tabatabaie, Khakpai, & Zarrindast, 2015) memory consolidation.

Interestingly, the cognition enhancing effects of 5-HT_4_ receptor activation have also been demonstrated in the chronic corticosterone model of anxiety/depression (Darcet, Gardier, David, & Guilloux, 2016), suggesting that this may be a promising strategy for treating the cognitive impairments associated with stress-related disorders. Chronic administration of the 5-HT_4_ receptor agonist RS67333 restored corticosterone-induced deficits on a broad range of tests of learning and memory, in contrast to chronic fluoxetine, which had a much narrower profile of effects. The 5-HT_4_ receptor agonist RS67333 has also been shown to restore emotional memory performance in a passive avoidance test in the Flinders sensitive line rat model of depression (Eriksson et al., 2012). Together these findings suggest that 5-HT_4_ receptor agonists may be a potential target for the improvement of the cognitive and emotional processing deficits associated with depression.

In contrast to the clear pro-cognitive effects of 5-HT_4_ receptor activation, the effect of 5-HT_4_ receptor antagonism is less well characterised and findings are mixed. Whilst the antagonists SDZ205557 and RS67532 have been shown to impair olfactory association memory formation and passive memory formation (Galeotti, Ghelardini, & Bartolini, 1998; Marchetti, Dumuis, Bockaert, Soumireu-Mourat, & Roman, 2000), other studies report no effect of 5-HT_4_ receptor antagonism on memory processes (Moser et al., 2002; Orsetti et al., 2003). It has been suggested that this may be due to a low level of behaviourally-mediated exogenous serotonin release, which would lessen the effects of receptor blockade compared with receptor activation (Orsetti et al., 2003). Likewise, 5-HT_4_ receptor knock-out mice do not have clear behavioural deficits in learning and memory (Segu et al., 2010). However, these animals do show an impairment in long-term spatial memory on the Morris water maze following scopolamine administration at a dose ineffective in wild type controls, suggesting that developmental adaptations in the cholinergic system may compensate for the absence of 5-HT_4_ receptors under baseline conditions (Segu et al., 2010).

The memory enhancing properties of 5-HT_4_ receptor activation are often attributed to a facilitation of central cholinergic activity. This is supported by studies demonstrating that 5-HT_4_ receptor agonists enhance the brain ACh output under both baseline conditions (Consolo et al., 1994) and during mnemonic demands (Mohler et al., 2007), and that 5-HT_4_ receptor agonists reverse the cognitive impairments induced by muscarinic antagonists (Cachard-Chastel et al., 2008; Fontana et al., 1997; Lo et al., 2014; Marchetti-Gauthier et al., 1997; Moser et al., 2002). For example, acute 5-HT_4_ receptor activation reverses the impairments in spatial learning and memory that are induced by atropine and scopolamine on the Morris water maze (Fontana et al., 1997; Lo et al., 2014) and the deficits in passive avoidance behaviour that are induced by scopolamine on the passive avoidance test (Galeotti et al., 1998; Lo et al., 2014). More specifically, local infusion of the 5-HT_4_ receptor agonist RS67333 into the NbM enhances the acquisition and consolidation of place recognition memory, which has been suggested to be due to a potentiation of the activity of the cholinergic NbM-cortical pathways (Orsetti et al., 2003; although see Penas-Cazorla & Vilaro, 2015). Further support for the idea that the memory-enhancing effects of 5-HT_4_ receptor activation are mediated via an interaction with cholinergic systems comes from studies that demonstrate that acetylcholinesterase inhibitors (AChEI) potentiate the effects of 5-HT_4_ agonists on cognition (Cachard-Chastel et al., 2007; Lamirault, Guillou, Thal, & Simon, 2003; Mohler et al., 2007; Moser et al., 2002). For example, sub-efficacious doses of the 5-HT_4_ receptor agonist prucalopride and the AChEI donepezil reverse scopolamine-induced deficits in spatial learning and memory when given in combination (Cachard-Chastel et al., 2007).

## Establishing the effects of 5-HT_4_ receptor activation in humans

Taken together, this preclinical evidence suggests that the 5-HT_4_ receptor may be a promising target for the treatment of depression and cognitive disorder in humans. However, there have been relatively few studies in humans to establish the translation of these effects, and to characterise the profile of 5-HT_4_ receptor activation on human cognition and emotion.

There is some initial evidence to support a role for the 5-HT_4_ receptor in the neurobiology of depression. Healthy individuals with a family history of depression have been shown to have lower 5-HT_4_ receptor binding in the striatum, and this reduction is most pronounced in people with more than one first degree relative with a history of depression (Madsen et al., 2014). Conversely, an increase in the density and functionality of 5-HT_4_ receptors in cortical and striatal areas has been reported in an analysis of post-mortem brains from depressed violent suicide victims (Rosel et al., 2004). Polymorphisms in the splice variant region of the gene encoding the 5-HT_4_ receptor have been reported to be associated with unipolar depression (Ohtsuki et al., 2002), although this was not confirmed in a recent genome wide association study (Wray et al., 2018). An involvement of the 5-HT_4_ receptor in human memory is also supported by a PET study that reported an inverse correlation of 5-HT_4_ receptor binding in the hippocampus and performance on the Auditory Verbal Learning Task (Haahr et al., 2013). Interestingly, the direction of this effect is opposite to the memory enhancing effects of 5-HT_4_ receptor agonism seen in preclinical studies, which may be due to the binding potential representing a composite measure of receptor density and affinity and/or that lower 5-HT_4_ receptor binding could also be reflective of higher chronic endogenous serotonin levels (Haahr et al., 2014).

These divergent correlational findings highlight the importance of further investigation in humans using an experimental medicine approach. Establishing the cognitive effects of compounds targeting the 5-HT_4_ receptor is particularly important in light of the limited predictive validity of animal models and the disappointing failure to predict the efficacy of pro-cognitive and antidepressant drugs in humans from drug discovery studies in rodents (Hyman, 2012; Millan et al., 2012). We have previously demonstrated that acute administration of clinically active antidepressants in healthy volunteers and depressed patients produces changes in information processing that can be measured using behavioural and neuroimaging paradigms (Godlewska & Harmer, 2020). Importantly, these changes are predictive of later therapeutic effects and these measures can therefore be used as surrogate markers of clinical effects to screen and understand the mechanisms of candidate agents early in development. Testing the effects of compounds targeting the 5HT_4_ receptor in humans using this approach could help clarify the role of the 5HT_4_ system in cognition and emotion and translate the potentially promising results from preclinical models to clinical application.

Experimental medicine often takes a stepwise approach, first testing the effects of the compound in healthy participants to gain information on relevant dosing, duration of treatment and specific cognitive and emotional processes which are sensitive to this manipulation. This information can then be used to guide the most appropriate design for testing in clinical groups including those with depression, anxiety and cognitive impairment. Again, experimental medicine markers (such as measures of learning or emotional processing) can be useful prior to clinical testing of efficacy (symptom improvement) to characterise likely mechanisms, target and patient groups. Such a stepwise approach may improve decision making about a compound's development and reduce the high failure rate associated with drug development in this area (Wong, Siah, & Lo, 2019).

Until recently, further elucidation of the role of the 5-HT_4_ receptor in humans has been difficult because of the lack of suitable compounds to manipulate 5-HT_4_ receptor function. The development of 5-HT_4_ receptor agonists for use as antidepressants in humans is complicated by the important role played by the 5-HT_4_ receptor in the periphery, in particular in the gastrointestinal tract and the heart, and the potential for peripheral side effects. Indeed, two 5-HT_4_ agonists, cisapride and tegaserod, that were developed for the treatment of gastrointestinal problems have both been restricted in use or withdrawn due to concerns about cardiac side effects. However, the particular concern with these drugs was risk of ventricular arrhythmias, which is probably due to their interaction with the human ether-a-go-go related gene potassium channel (De Maeyer, Lefebvre, & Schuurkes, 2008). Newer, more selective partial agonists, such as prucalopride which is licensed for the treatment of constipation, have good cardiac safety profiles. Prucalopride has good brain penetration (Johnson et al., 2012), has pro-cognitive and antidepressant-like effects in animal models (Cachard-Chastel et al., 2007; Lucas et al., 2007) and therefore offers a unique opportunity to investigate the role of the 5-HT_4_ receptor in human cognition.

We recently explored the effect of a single dose of prucalopride in healthy volunteers and found consistent pro-cognitive effects across measures of learning memory including declarative memory, reward learning and emotional encoding (Murphy, Wright, Browning, Cowen, & Harmer, 2019). These findings replicate the findings in preclinical studies and support further clinical assessment of these compounds in depression and for disorders involving memory impairment. In line with data in animal models that 5HT_4_ agonists may be beneficial in depression, and also in combination with an SSRI, we are currently exploring the effects of a 5HT_4_ partial agonist (PF-04995274) on emotional and cognitive function in unmedicated depressed patients (NCT03516604) and patients who have not showed a sufficient response to current SSRI treatment (NCT03515733). This kind of experimental medicine approach can provide early proof-of-concept evidence to support larger clinical trials, and may have value in selecting the best treatment, for the right subgroup of depressed patients, prior to full randomised clinical trials of efficacy.

## Conclusions

There has been much interest in the psychotropic effects of 5-HT_4_ receptor activation, stimulated by consistent evidence that 5-HT_4_ receptor agonists have antidepressant-like and pro-cognitive effects on a range of animal models. However, the translation of these effects to humans has been slow, and, to date, no 5-HT_4_ receptor agonists have been licensed for any indication other than gastrointestinal disorders. Whilst there had been some concern about the safety of 5-HT_4_ receptor agonists, due to this receptor's important roles outside of the central nervous system (for example in the heart), prucalopride has proved to be a safe approach to the treatment of chronic constipation. Experimental medicine offers a cost-effective way to efficiently translate these effects into humans and explore the possibility that 5-HT_4_ receptor activation might be a useful adjunct to antidepressant treatment, both to speed the onset of clinical antidepressant effects and to target the cognitive symptoms that are not well addressed by current treatments.
